# *In virio* SHAPE analysis of tRNA^Lys3^ annealing to HIV-1 genomic RNA in wild type and protease-deficient virus

**DOI:** 10.1186/s12977-015-0171-7

**Published:** 2015-05-16

**Authors:** Elias Seif, Meijuan Niu, Lawrence Kleiman

**Affiliations:** Lady Davis Institute for Medical Research and McGill AIDS Centre, Jewish General Hospital, Montreal, QC H3T 1E2 Canada; Department of Medicine, McGill University, Montreal, QC H3A 1A1 Canada

**Keywords:** HIV-1, tRNA^Lys3^, SHAPE, annealing, Gag, NCp7

## Abstract

**Background:**

tRNA^Lys3^ annealing to the viral RNA of human immunodeficiency virus type-1 (HIV-1) is an essential step in the virus life cycle, because this tRNA serves as the primer for initiating reverse transcription. tRNA^Lys3^ annealing to viral RNA occurs in two steps. First, Gag promotes annealing of tRNA^Lys3^ to the viral RNA during cytoplasmic HIV-1 assembly. Second, mature nucleocapsid (NCp7), produced from the processing of Gag by viral protease during viral budding from the cell, remodels the annealed complex to form a more stable interaction between the viral RNA and tRNA^Lys3^, resulting in a more tightly bound and efficient primer for reverse transcription.

**Results:**

In this report, we have used *in virio* SHAPE analysis of both the 5´-untranslated region in HIV-1 RNA and the annealed tRNA^Lys3^ to determine structural differences of the annealed complex that occur between protease-negative (Pr-) and wild type viruses. Our results indicate that the weaker binding of tRNA^Lys3^ annealed by Gag in Pr- virions reflects both missing interactions of tRNA^Lys3^ with viral RNA regions in the upper PBS stem, and a weaker interaction with the internal stem-loop found within the unannealed primer binding site in viral RNA.

**Conclusions:**

We propose secondary structure models for the tRNA^Lys3^/viral RNA annealed complexes in PR- and wild type viruses that support the two-step annealing model by showing that Gag promotes a partial annealing of tRNA^Lys3^ to HIV-1 viral RNA, followed by a more complete annealing by NCp7.

**Electronic supplementary material:**

The online version of this article (doi:10.1186/s12977-015-0171-7) contains supplementary material, which is available to authorized users.

## Background

Following infection of a host cell by HIV-1, the viral RNA genome (vRNA) is reverse transcribed into a double-stranded DNA, which then integrates into the host cell’s genome. Reverse transcription is initiated from a primer tRNA^Lys3^ that was previously annealed during viral assembly to an 18 nt sequence in the viral RNA termed the primer binding site (PBS). The 18 nt PBS (nt 182–199) is found immediately downstream of the unique 5´ (U5) region in viral RNA, and is complementary to the 3´ terminal 18 nt of tRNA^Lys3^. Annealing to the PBS sequence is required for tRNA^Lys3^ annealing [1–3]. Two other regions in the 5´-untranslated region (5´-UTR) upstream of the PBS, the poly(A) loop and the C-rich region, have also been implicated in interacting with complementary sequences in the 3´-half of tRNA^Lys3^. They were first proposed in the case of an HIV-1 MAL isolate that belongs to sub-type A by Marquet group [4, 5]. While the presence of these interactions in sub-type B isolates (HXB2 and NL4.3) was controversial [6], the Weeks group was able to detect these additional interactions in wild type virions using SHAPE [7]. An additional viral RNA sequence upstream of these sequences, termed the primer activation site (PAS), was also reported to interact with complementary sequences in the 5´-half of the TΨC arm [8] in tRNA^Lys3^, but this interaction appears to occur only during the initiation of reverse transcription [9].

The annealing of tRNA^Lys3^ to viral RNA is a multi-step process, promoted by both the viral Gag precursor, and later, after viral protein processing, by mature nucleocapsid (NCp7). tRNA^Lys3^ annealing to viral RNA *in vitro* at 37 °C does not occur at a wide range of RNA concentrations unless either Gag or NCp7 is present [10, 11]. Furthermore, specific mutations within nucleocapsid sequences in Gag can reduce tRNA^Lys3^ annealing *in vivo* in protease-negative viruses up to 80 %, independently of any effect such mutations might have on viral genomic RNA packaging [12]. Gag promotes the first step in annealing within a cytoplasmic annealing complex representing an early HIV-1 assembly intermediate, whose composition includes Gag, GagPol, lysyl-tRNA synthetase (LysRS), viral RNA, and tRNA^Lys3^ [13]. The binding of both lysyl-tRNAs and non-lysyl tRNAs to this complex takes place prior to budding, and most likely at the site of translation of viral proteins, since the relative concentration of non-lysyl tRNAs in the virus match the codon usage of viral mRNA, not cellular mRNA [14, 15]. However, The concentration of tRNA^Lys^ relative to non-lysyl tRNAs is also increased 8–10 fold over that in the cytoplasm due to a specific interaction of Gag with the major tRNA^Lys^ binding protein, lysyl-tRNA synthetase [13].

Compared to NCp7-annealed tRNA^Lys3^, Gag-annealed tRNA^Lys3^ has a 2/3 reduced ability to initiate reverse transcription [16], and binds more weakly to viral RNA [17]. Thus, using total viral RNA extracted from wild type (Pr+) or protease-negative (Pr-) viruses, the weaker binding of tRNA^Lys3^ was shown by the ability of an *in vitro* reverse transcript from a primer upstream of the primer binding site to displace tRNA^Lys3^ annealed by Gag in a Pr- virus, but unable to displace tRNA^Lys3^ annealed by NCp7 in a Pr+ virus. Furthermore, in that report, we showed that weaker binding to viral RNA, and the reduced ability of Gag-annealed tRNA^Lys3^ to function as a primer for reverse transcription, can be rescued by a transient exposure to purified NCp7 of total viral RNA isolated from Pr(−) virions [17]. This suggests that while Gag may initially anneal tRNA^Lys3^ to the viral RNA, after viral protein processing, which may occur during or after viral budding [18], exposure of the annealed complex to NCp7 may fine-tune the annealed tRNA^Lys3^ to produce an annealed tRNA^Lys3^ that will function more efficiently as a primer.

The aim of this study is then to elucidate the structural differences between Gag- and NCp7-mediated tRNA^Lys3^ annealing within the viral particle. To examine how tRNA^Lys3^ anneals to HIV-1 RNA, we compared the structural interactions that occur between tRNA^Lys3^ and viral RNA in protease-negative (Pr-) and wild type (Pr+) virions, using *in virio* SHAPE (selective 2´-hydroxyl acylation analyzed by primer extension). This method was developed by the group of K. Weeks [19], and used to predict the secondary structure of the annealed tRNA^Lys3^/viral RNA complex in wild type virions, based upon the reactivity of individual nucleotides (nts) in the viral RNA with the acylating agent NMIA (N-methylisotoic anhydride). NMIA reacts with the ribose 2´-hydroxyl group in flexible, unpaired, nucleotides. In this work, we have used SHAPE to measure the reactivity toward NMIA of nts in both viral RNA and annealed tRNA^Lys3^, within both Pr- and wild type virions. Our results indicate that the weaker binding of tRNA^Lys3^ annealed by Gag in Pr- virions reflects missing interactions of tRNA^Lys3^ with the upper PBS stem, and an altered interaction of tRNA^Lys3^ with the internal stem loop found within the unannealed PBS sequence.

## Results

### *In virio* SHAPE reactivities of the 5´-UTR from wild type and protease-negative HIV-1

**Fig. 1:**
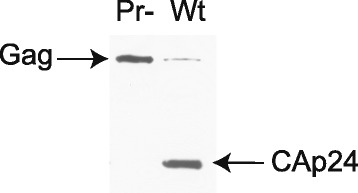
Gag processing in wild type and Pr- viral particles. Wild type and Pr- viral particles produced from transfected 293 T cells were lysed, and a Western blot of the viral lysates was probed with anti-CAp24, as described in Methods

**Fig. 2:**
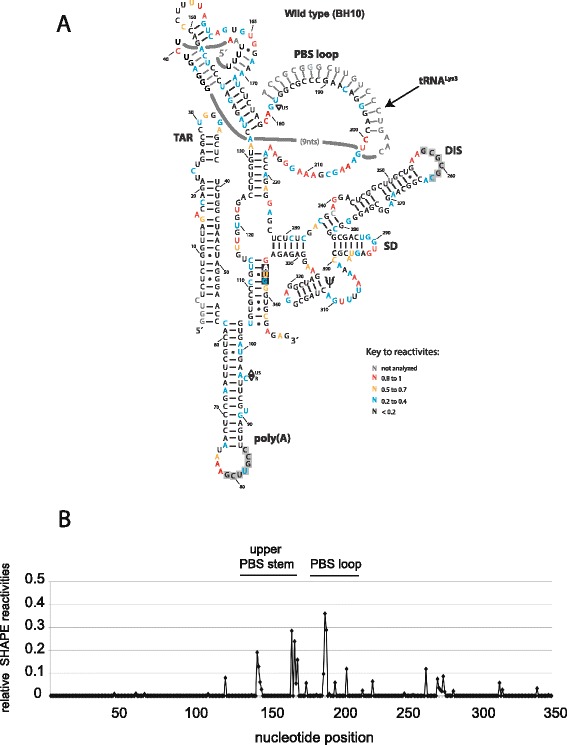
Secondary structure of the HIV-1 5´-UTR in wild type and Pr- HIV-1. (**a**) Secondary structure model for HIV-1 wild type 5´-UTR. The secondary structure model was built using RNAstructure software based on the reactivities determined in this study. The low reactivities seen for nucleotides G79 to C85 (grey box) were proposed to result from an interaction with downstream sequences G443 to C449 [19]. Similarly, The low reactivities of the grey-boxed nucleotides in the DIS loop (nts 257–262) have been proposed to result from pairing with a homologous viral RNA molecule in the viral RNA dimer. The AUG start codon is boxed in black. (**b**) The difference in SHAPE reactivities of Pr- viral 5´-UTR relative to the reactivities of wild type 5´-UTR. The difference in SHAPE reactivities between Pr- and wild type 5´-UTRs were calculated by subtracting the sum of the standard deviations at each nt position from the absolute value of the difference of reactivities at every position for each data set (|(reactivity^Pr-^)-(reactivity^wt^)|-(Std^Pr-^ + Std^wt^)). A zero value was assigned to positions where the result was negative, implying that the error is higher than the difference in reactivities. Positive values indicate that the reactivity toward NMIA at the specified nucleotides is higher in the Pr- virions. The calculations and the graph plot were drawn using Excel from the Microsoft Office Package

Extracellular wild type BH10 (Pr+) and protease-negative BH10-Pr- (Pr-) were produced from transfected 293 T cells, and purified on a sucrose-gradient as described in Methods. Figure 1 shows a Western blot of viral lysate probed with anti-CAp24, and verifies the absence of Gag processing in Pr- virus. The viral RNA was modified within the intact viral particles by NMIA, and then extracted from the viral particles. The reactivity toward NMIA at each nucleotide (nt) position was determined by SHAPE. The average reactivity at each position and the standard deviations were calculated for nucleotides 5 to 348, using three independent experiments for both wild type and Pr- viral RNA. Nucleotides (nts) 1 to 4 and 349 to 406 were not analyzed due to ambiguous alignment at the 5´ and 3´ ends of the RNA. We refer to nts 5–348 as the 5´ untranslated region (5´-UTR) of the viral RNA, although it also includes the first 14 nts coding for Gag. In Fig. 2a, we show the secondary structure of wild type 5´-UTR predicted using the program RNAstructure, which combines nt reactivities obtained using *in virio* SHAPE with the proposed best thermodynamically stable structures, as described in Methods. The secondary structure model is similar to the previously published model for wild type HIV-1 by Weeks’s group [20], with a minor difference being that the U5:AUG stem is extended by three additional base pairings in our model. The predicted interaction of tRNA^Lys3^ with the viral RNA shown in Fig. 2a, while derived from our reactivity data, is based on a previously proposed model for how tRNA^Lys3^ interacts with the viral RNA [19]. In addition to the PBS, tRNA^Lys3^ will also bind to two other regions in the upper PBS stem of viral RNA, termed the C-rich and poly(A) loop regions. SHAPE reactivities and the predicted secondary structure model of the 5´-UTR within Pr- viruses are similar to those of wild type viruses, except in the regions that include the upper PBS stem and the PBS loop (see Fig. 2a). This is shown in Fig. 2b, in which the SHAPE reactivities of Pr- viral 5´-UTR are plotted relative to the reactivities of wild type 5´-UTR.

### Structural differences within the viral RNA/tRNA^Lys3^ annealed complex

**Fig. 3:**
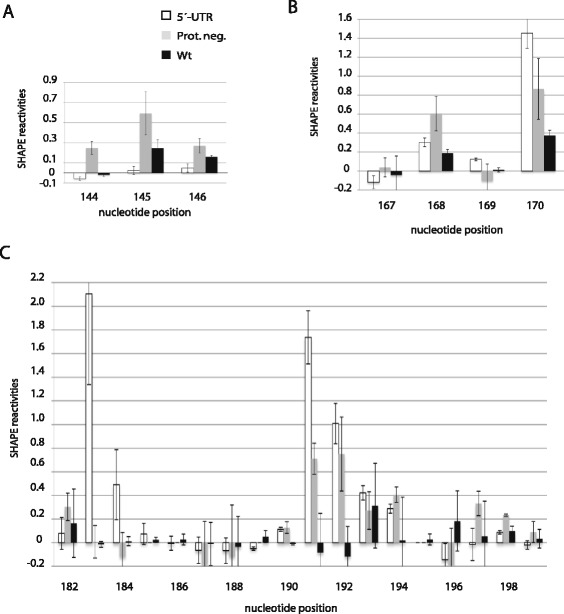
The SHAPE reactivities for regions of annealing in the HIV-1 5´-UTR. SHAPE reactivities for *in vitro* transcribed unannealed HIV-1 5´-UTR (white), and viral RNA found in Pr- (grey), and wild type (black) viral particles are shown for sequences containing (**a**) nts 144 to 146, (**b**) nts 167 to 170, and (**c**) nts 182 to 199. The results shown are the averages of three readings at each nucleotide position for 5´-UTR, Pr- and wild type. The errors shown in the graph correspond to the standard deviations calculated at each nucleotide position

**Fig. 4:**
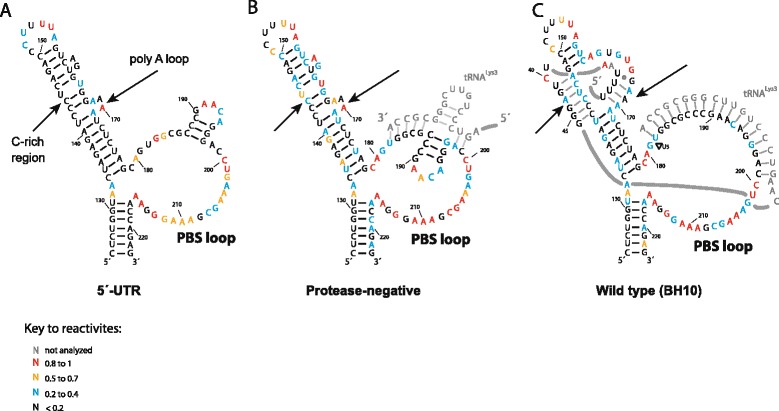
Models for tRNA^Lys3^ binding to regions of annealing in the HIV-1 5´ UTR, based on the reactivities shown in Fig. 3. Secondary structure models of the PBS stem-loop from (**a**) *in vitro* transcribed unannealed HIV-1 5´-UTR, and from RNA present in (**b**) Pr- and (**c**) wild type virions. Only sequences of tRNA^Lys3^ involved in the annealing to viral RNA are shown in the figures

To better understand the significance of the observed differences in reactivities shown in Fig. 2b in the context of tRNA^Lys3^ annealing, we compared *in virio* SHAPE reactivities in these regions for wild type and Pr- virions with the SHAPE reactivities of an unannealed *in vitro* transcribed 5´-UTR of HIV-1. We previously described the production and characteristics of this construct [20]. Figure 3 shows the individual SHAPE reactivities for each nucleotide in these regions, while Fig. 4 shows secondary structure models of the PBS stem-loop regions predicted using the RNAstructure program for the unannealed *in vitro* transcribed 5´-UTR, and viral RNAs found in Pr- and wild type virions. The unannealed PBS sequence itself contains an internal stem loop, and other nucleotides with low reactivities within the larger PBS loop suggest some tertiary interactions that constrain the flexibility of these nucleotides. As seen in Figs. 3c and 4c, all of the PBS nucleotides show relatively low SHAPE reactivity in the annealed viral RNA from wild type viruses (Figs. 3c and 4b). In RNA from the Pr- virus significantly higher reactivities can be seen for internal loop nucleotides 191 and 192, suggesting a less efficient annealing of tRNA^Lys3^ to these sequences by Gag. Nucleotides 193 and 194 show low reactivities in unannealed, Pr-, and Pr + RNAs, and the differences between all three samples are not significant. These nucleotides are probably stabilized in a conformation unfavorable for modification by NMIA, even if they are not involved in a Watson-Crick base pair. In Fig. 4b, we propose a structural model for the Pr- primer binding site reflecting the observed SHAPE reactivities. To verify that the decrease in reactivities of nucleotides 183 and 184 within the PBS sequence of the Pr- viral RNA does not result from tRNA^Lys3^ annealing in the absence of Gag, we incubated *in vitro* transcribed 5´-UTR viral RNA and tRNA^Lys3^ (1 to 10 ratio) in the absence of Gag for 30 min at 37 °C, and performed SHAPE experiments. The reactivities within the PBS sequence remained similar to that of unannealed 5´-UTR RNA (see Additional file 1).

As shown in Figs. 3a,b and 4c, the upper PBS stem in the viral RNA includes two additional sites that interact with the tRNA^Lys3^. The first includes nts C142-A147 and is referred to as the C-rich region (Figs. 3a and 4). In unannealed viral RNA, these nts show low reactivities, as they are part of the upper PBS stem. Similar low reactivities are found in this region in viral RNA from wild type viruses, since the C-rich region interacts with part of the variable loop and anticodon stem of tRNA^Lys3^ (Fig. 4c). However, as shown in Fig. 3a, three nts (144–146) of Pr- mutant show an increase in reactivities compared to either unannealed viral RNA or viral RNA from wild type viruses, suggesting a weaker interaction in this region. The second interacting site in viral RNA includes nts G167-A170 and is referred to as the poly(A) loop. This site interacts with the U-rich anticodon loop of tRNA^Lys3^. Nts 168 and 170 show higher reactivities in the Pr- mutant than in wild type virus (Fig. 3b). These observations suggest that the interactions between tRNA^Lys3^ and vRNA are also weakened in the Pr- virus. To verify that the observed differences result from tRNA/vRNA interactions and not from differences in RNA-protein interactions, we have treated both Pr + and Pr- viral particles with proteinase K prior to RNA modification using NMIA (*ex virio*) and performed SHAPE analysis. The complete data sets are shown in Additional file 2. Several changes in reactivities were observed in the upper PBS stem and the PBS loop (nts 130 to 217). For Pr+, changes occurred at nucleotides 136,137,147, 151, 156, 172, 178, 180, 181, 203, 206 and 207. For Pr-, changes occurred at nucleotides 137, 141, 156, 157, 159, 166, 171, 172, 177, 181, 182, 200, 203 and 204. However, the results presented in Additional files 3 and 4 show that *ex virio* and *in virio* shape reactivities are similar for the nucleotides we analyzed to establish our hypothesis, and support our assumption that the observed changes between Pr + and Pr- are due to an altered tRNA^Lys3^ annealing to viral RNA.

### SHAPE analysis of annealed tRNA^Lys3^*in virio*

**Fig. 5:**
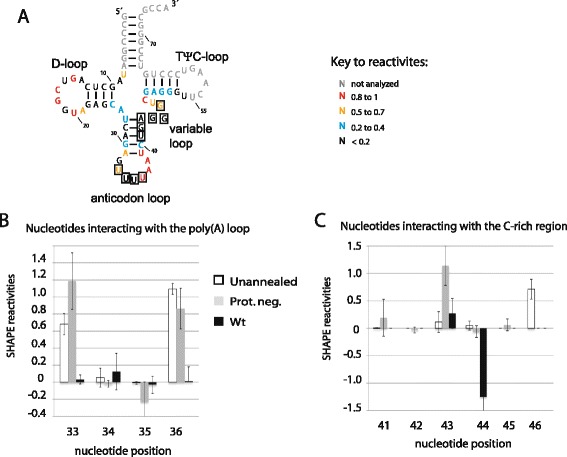
(**a**) SHAPE reactivities of the *in vitro* transcribed tRNA are shown on the secondary structure model of tRNA^Lys3^. Boxed nucleotides are implicated in annealing to viral RNA. SHAPE reactivities of tRNA^Lys3^ nts involved in annealing are shown in (**b**) for the anti-poly(**a**) region, and in (**c**) for the anti C-rich region. Reactivities from unannealed in vitro transcribed tRNA, annealed tRNA from wild type and Pr- virions are shown in white, grey and black respectively. The results shown are the averages of three readings at each nucleotide position. The errors correspond to the standard deviations calculated at each nucleotide position

Our conclusions thus far concerning the interactions occurring between viral RNA and tRNA^Lys3^ are based solely on SHAPE analysis of viral RNA. To investigate the validity of these conclusions, we also determined the SHAPE reactivities of the annealed tRNA^Lys3^ within wild type and Pr- viral particles. In this procedure, we eliminated non-annealed tRNAs present in the virion by using columns that retain only RNAs larger than 200 nts after extracting the NMIA-modified RNA from the viral particles. The annealed tRNAs were released from the viral genomic RNA by heating to 70 °C for 5 min, and poly(A) tails were added to the tRNA as described in Methods. The reverse transcription was primed by an oligomer specific to tRNA^Lys3^ at the 3´ end. As shown in Fig. 5, SHAPE reactivities were determined for nts 7–55 of the tRNA. The reactivities for nucleotides 1 to 6 and 55 to 76 were not determined because the primer used for the primer extension reaction includes the 3´ terminal nucleotides of the tRNA, and because the sequence alignment close to the 5´ and 3´ ends is ambiguous. Consequently, SHAPE reactivities for the 3´ terminal 18 nts of tRNA^Lys3^, the sequence that binds to the PBS, could not be calculated. For comparative studies we also determined SHAPE reactivities of an in vitro transcribed tRNA^Lys3^. Several stops were noticed in the modified and the unmodified reverse transcription reactions of the *in virio* tRNAs from wt and Pr- viral particles, but not in the *in vitro* transcribed tRNA. These stops correspond to modified nucleotides 10, 16, 20, 37, 47, and 53. These nucleotides were marked as not analyzed. They are shown in grey in the figures, and given a −999 value in the reactivities datasets (see Additional file 2).

The region including nucleotides U33-U36, which is part of the anticodon loop in annealed tRNA^Lys3^, is referred to in Fig. 5 as the anti-poly(A) loop, because it interacts with the poly(A) loop of viral RNA. The anti-poly(A) loop region from annealed tRNA^Lys3^ in wild type virions is non-reactive to NMIA, indicating that it is annealed in these virions (Fig. 5). However, for annealed tRNA^Lys3^ from Pr- viruses and for the *in vitro* transcribed tRNA, U34 and U35 retain low reactivities (<0.2), while U33 and U36 have high reactivities (see Fig. 5). This would suggest that U34 and U35 adopt a conformation that inhibits their modification by NMIA, although we cannot exclude the possibility of an intermolecular pairing. In Fig. 4b, we see that in the poly(A) area within viral RNA, the G167 that could have paired with the highly reactive tRNA^Lys3^(U33) has in fact low reactivity, presumably because it forms a base pair within the viral stem. Similarly, the low reactivity of tRNA^Lys3^(U34) coupled with the high reactivity of A168 in viral RNA also suggests that these two nts are not base- paired, and that the low reactivity of tRNA^Lys3^(U34 and U35) in the tRNA is a result of a failure of NMIA modification as discussed above. Low reactivities for both tRNA^Lys3^(U35) and vRNA(A169) suggest a possible base pair, but the high reactivities of both A170 in viral RNA and U36 in tRNA^Lys3^ suggest the absence of base pairing. Thus, 3 of the 4 potential base pairs in this region of interaction between viral RNA and tRNA^Lys3^ are absent in the Pr- virus.

Low reactivities in the C-rich region of viral RNA (nts 142–147) could result from maintaining the upper PBS stem, or could result from annealing with a complementary region in tRNA^Lys3^, referred to in Fig. 5 as the anti-C-rich region (tRNA^Lys3^ (41–46)). U145 is the only nucleotides that would be free if the annealing is weakened or does not occur (see Fig. 4a). As expected by our conclusions, the nucleotides U145 show a significant increase in reactivity in the Pr- viral RNA (Fig. 3a). In support of this interpretation, we find that nts 41–46 in tRNA^Lys3^ do have low reactivity, i.e., are base-paired. However in Pr-, one out of six nucleotides from the anti-C-rich region of the tRNA show high reactivities, 1.1 for A43 (Fig. 5). The limited increase in reactivities for the Pr- results from the alternative structure that the unannealed regions would adopt. These observations support the conclusion that in Pr- mutant, tRNA^Lys3^ annealing to the upper PBS stem is missing.

## Discussion

Previous studies have indicated that tRNA^Lys3^ annealed either by Gag *in vitro*, or within a Pr- virus, is less tightly bound to the viral genomic RNA, and is a less efficient primer for reverse transcriptase, than tRNA^Lys3^ annealed either *in vitro* with mature nucleocapsid (NCp7) or in wild type viruses [17, 18]. We used *in virio* SHAPE analysis to investigate these differences while inducing minimal changes in the RNA structure during isolation of RNA from viruses. The results reported here show structural differences within the tRNA^Lys3^/viral RNA complex isolated from wild type or Pr- viruses that correlate with these findings. Although some positions show high standard deviations, all our experiments were repeated three times independently, and showed a similar trend in all cases. The proposed models are further supported by the changes in NMIA reactivities occurring only in the interacting regions of viral RNA and the tRNA^Lys3^ (Figs. 2b, 3 and 4), i.e. on the complementary strands of the upper PBS stem, and between the viral RNA and the complementary region in the tRNA. Our models corroborate earlier observations showing that annealing is weaker in protease-negative virions than in wild type virions (17, 18). Our results indicate that the weaker binding of Gag-annealed tRNA^Lys3^ results from a poor ability of Gag to anneal tRNA^Lys3^ sequences to the internal loop present within the unannealed PBS sequence, and to the C-rich and poly(A) regions in viral RNA upstream of the PBS that are also involved in annealing in wild type viruses. SHAPE reactivities obtained for both the annealed regions in viral RNA and the C-rich and poly(A) regions of annealed tRNA^Lys3^ were found to be complementary, but for technical reasons, the SHAPE reactivities of the 3´-terminal 18 nts of tRNA^Lys3^ could not be obtained and compared with the PBS SHAPE reactivities.

In this work, the differences in acylation sensitivity found in Pr + and Pr- samples is assumed to reflect differences in RNA backbone structure, and not different abilities of protein bound to the RNA to inhibit or promote access to the acylation reagent. To support this assumption, we performed SHAPE analysis on de-proteinized Pr + and Pr- viral RNA, and found that the nucleotides we analyzed did not show any significant difference in SHAPE reactivity in absence or presence of the proteins. In fact, the effect of proteins on NMIA modification was previously studied by Kevin Weeks’s group for the first 5´- 990 nts of the HIV-1 genome, and done both *in virio* and *ex virio* [19], SHAPE reactivity patterns for the *in virio* and protein-free *ex virio* states were very similar for the major length of the viral RNA. They also examined more specifically the effect of NCp7 binding on SHAPE reactivities *in virio* by exposing virions to a reagent that inhibited NCp7 binding to the viral RNA. They found distinct regions in the viral RNA that bound to NCp7, which depending upon the nature of the binding, could cause increases or decreases in NMIA reactivity of individual nts. However, none of the nts demonstrated here to be involved in tRNA^Lys3^ annealing showed binding to NCp7 in their study, and in fact, these nts showed similar reactivities to NMIA both *in virio* and *ex virio*. Furthermore, to support our interpretation of the changes observed, we have shown that changes in NMIA reactivities noticed in the annealing areas on the viral RNA are accompanied by similar changes in the complementary nucleotides on the tRNA^Lys3^ molecule.

The inability of Gag to anneal tRNA^Lys3^ to regions in viral RNA upstream of the PBS may be due to a limited ability of nucleocapsid sequence within Gag to denature RNA duplexes. It has been observed previously that Gag and mature NCp7 destabilize RNA structures in different propensities [21, 22]. NMR studies have shown that NCp7 protein has a dynamic structure that helps binding to nucleic acids with different sequence and structure [23], and that the conformation of nucleocapsid sequence within Gag could be influenced by the overall Gag conformation. For example, the matrix (MA) domain in Gag has been shown to modulate Gag’s nucleic acid chaperone activity via binding to inositol phosphate (IP) in the membrane [24]. In absence of IP, the MA domain inhibits Gag’s tRNA annealing capacity. NC’s conformation and annealing ability could also be limited by its local interaction with neighboring Gag sequences.

While nucleocapsid sequences within Gag can promote a relatively weak interaction of the terminal 18 nts of tRNA^Lys3^ with the PBS in viral RNA, it appears that only mature NCp7 promotes the interactions of two regions within the anticodon stem-loop of tRNA^Lys3^ with nucleotides 142–147 and 167–170 of the upper PBS stem. The *in vitro* interaction of NCp7 with tRNA^Lys3^ has been shown to destabilize the acceptor stem, the T-stem-loop, and the anticodon stem-loop [25, 26], and it would be of interest to determine if Gag is able to destabilize the anticodon stem-loop as efficiently as NCp7.

The difficulty of bringing cytoplasmic tRNA^Lys3^ to an 18 nt PBS sequence within a structurally complex >9 kb viral genomic RNA may explain the need for a multistep annealing mechanism, even if the initial cytoplasmic binding of the tRNA^Ly3^ to viral RNA is weak relative to annealing by mature NCp7. An early cytoplasmic HIV-1 assembly intermediate composed of unprocessed Gag and GagPol and viral RNA will promote this initial annealing through its molecular organization [13], its ability to selectively concentrate tRNA^Lys3^ at the cytoplasmic site of viral assembly through a specific Gag/LysRS interaction [27], and its selective binding to RNA sequences close to the PBS [23, 28]. These properties of this complex will be lost upon viral protein processing.

The cytoplasmic annealing of tRNA^Lys3^ to viral RNA by Gag appears to serve another function in addition to preparing tRNA^Lys3^ to act as a primer for reverse transcription. Thus, it has been reported that annealing of tRNA^Lys3^ to the PBS aids in promoting a conformation change in the 5´-untranslated region of viral RNA that promotes viral RNA dimerization [11, 20], a process required for efficient incorporation of viral RNA into the virus [29, 30].

## Conclusions

In conclusion, our study confirms the findings that in wild type HIV-1 subtype B virions, interactions occur between tRNA^Lys3^ and the upper PBS stem. We also found major differences in the interactions between viral RNA and annealed tRNA^Lys3^ from Pr- and wild type virions. These differences reveal the reasons why annealed tRNA^Lys3^ is less tightly bound in Pr- virions. These findings are consistent with the multi step annealing mechanism proposed previously.

## Methods

### Plasmids

SVC21.BH10 is a simian virus 40-based vector that codes for the wild type sequence of HIV-1 proviral DNA [31]. The protease-deficient mutant (Pr-) is an SVC21.BH10 plasmid coding for an inactive protease with an Asp25 to Arg25 mutation [32].

### Viral particle production and modification by NMIA

For each SHAPE reading, 24 cell culture plates (15 cm diameter) were inoculated with 6 million HEK 293 T cells per plate and grown in complete Dulbecco’s modified Eagles’s medium (DMEM). After overnight incubation, the cells were transiently transfected by wild type BH10 or BH10 Pr- plasmids using lipofectamine 2000 from Invitrogen according to the manufacturer’s recommendations. The supernatant was collected 48 h post-transfection, and filtered through 0.2 μm filters. The viral particles were pelleted in a Beckman Ti-45 at 35 000 rpm for 1 h. The pellets were then purified by 15 % sucrose onto a 65 % sucrose cushion by centrifugation in SW41 rotor at 26 500 rpm for 1 h. The layer of purified viral particles was collected and the virus was pelleted using Beckman SW41 rotor at 35 000 rpm for 1 h, in 1X TNE buffer (20 mM Tris–HCl pH 7.8; 100 mM NaCl, 1 mM EDTA). The viral particles were resuspended in 600 μl of NMIA reaction buffer (50 mM Hepes pH 8, 200 mM NaCl, 0.1 mM EDTA, and 10 % fetal bovine serum), and modified using NMIA for 50 min at 37 °C as described in [19]. For *ex virio* analysis, the viral particles were treated with proteinase K for 30 min at 37 °C prior to treatment with NMIA.

### Western blot

Prior to modification with NMIA, 2 % of the viral particles were mixed with protein gel loading dye (2X; 4 % SDS, 20 % glycerol, 10 % 2-mercaptoethanol, 0.004 % bromophenol blue and 0.125 M Tris HCl, pH 6.8), lysed by boiling, and run on a 10 % SDS-PAGE gel. Following gel transfer by electroblotting, the membrane was probed with mouse anti-CAp24 antibody (NIH AIDS Research and Reference Reagent Program).

### RNA purification and SHAPE analysis

The modified and control (unmodified) viral particles were lysed with 1 % SDS and Proteinase K treatment at room temperature for 20 min, followed by a phenol extraction as proposed by [19]. The RNA was purified from the lysate and concentrated using RNA clean & Concentrator-5 from Zymoresearch (Cat. #R1015). Equal amounts of RNAs (1 ρmole) were used for wild type and protease negative mutant. The RNA was reverse transcribed using Superscript III Reverse Transcriptase kit from Invitrogen (Cat. #18080-044), and a primer complementary to BH10 wild type sequences, nucleotides 382 to 406, labeled with VIC-fluorescent dye from Applied Biosystems (5´ VIC-CCTGGCCTTAACCGAATTTT TTCC 3´). The reactions were purified on Sephadex G-50 columns, ethanol precipitated, and resuspended in water. The ladder was prepared from purified unmodified viral RNA as previously described [20].

The products were resolved by capillary electrophoresis at the IRIC (Institut de Recherche en Immunologie et Cancérologie) genomic center. The data were analyzed and normalized as described in [20, 33] using peakScanner (freely available from ABI), FAST software (freely provided by the Jeffrey Glenn laboratory), and RNAStructure (freely provided by Dr. David Mathews laboratory). The unannealed RNA was produced by *in vitro* transcription from a BH10 template, and was analyzed by SHAPE as described previously [20].

### SHAPE analysis of annealed tRNA^Lys3^

The viral particles were produced and treated with NMIA as described for viral RNA. Following viral lysis, the RNA was treated with acidic phenol, and the aqueous phase was purified using Clean and Concentrate kit in conditions that retain RNAs larger than 200 nucleotides. The RNAs were heated to 70 °C for 5 min and then chilled on ice. Following a short spin at 12 000 rpm, the RNAs were treated with poly(A) polymerase using poly(A) tailing kit from Ambion (Cat# AM1350). The mix was treated with phenol, and the aqueous phase was purified on RNA clean and concentrate columns in conditions were RNAs larger than 17 nucleotides are retained. tRNA^Lys3^ was reverse transcribed as described for viral RNA in presence of VIC fluorophore labeled oligomer that includes 6 nucleotides specific to tRNA^Lys3^ and a poly U tail, 5´ VIC-TTTTTTTTTTTTTTTTGGCGCCCG 3´. The reverse transcription reaction was purified on Sephadex G25 column, ethanol precipitated, resuspended in water, and analyzed by capillary electrophoresis as described for viral RNA. The tRNA^Lys3^ ladder used for performing SHAPE analysis, was produced by sequencing a DNA template coding for tRNA^Lys3^ with a poly(A) tail, the same oligomer used for SHAPE analysis, and Sequenase^™^ version 2.0 DNA sequencing kit from USB (cat # 70770 1kt) according to the manufactuer’s recommendations for short readings.

### SHAPE analysis of unannealed 5´UTR in presence of non denatured tRNA^Lys3^

Three pmoles of *in vitro* transcribed 5´-UTR were incubated with thirty pmoles of tRNA^Lys3^, without heat denaturation, at 37 °C for 30 min, in 10 mM HEPES pH 8.0, 1 mM MgCl_2_, 10 mM NaCl, 150 mM KCl. SHAPE experiments were performed on the 5´-UTR RNA as described above.

## Additional files

Additional file 1:
**Comparison of SHAPE reactivities for viral RNA nucleotides 182-199 from 5´-UTR RNA, 5´-UTR RNA + tRNA** ^**Lys3**^ **, and Pr- virions.** SHAPE reactivities for *in vitro* transcribed unannealed HIV-1 5´-UTR (white), unannealed 5´-UTR + tRNA^Lys3^ (grey), and viral RNA found in Pr- (black) viral particles are shown for nucleodtides 182 to 199. The results shown are the averages of three readings at each nucleotide position. The errors shown in the graph correspond to the standard deviations calculated at each nucleotide position. The complete data sets are found in Additional file 2. Additional file 2:
**Complete data sets of SHAPE reactivities.** Complete data sets of SHAPE reactivities and the corresponding standard deviations for *in virio* Pr+, Pr-, and in vitro transcribed 5´UTR viral RNA in sheet 1; tRNA^Lys3^ from Pr + and Pr- viral particles and in vitro transcribed tRNA^Lys3^ in sheet 2, and *ex virio* Pr + and Pr- viral RNA in sheet 3. Additional file 3:
**Comparison of SHAPE reactivities for tRNA** ^**Lys3**^ **annealing sequences of Pr- viral RNA from intact** **(** ***in virio*** **) and proteinase K-treated viral particles (** ***ex virio*** **).** For *in virio* (grey), intact viral particles were treated with NMIA to modify the viral RNA within the viral particle, whereas for *ex virio* (white), the viral particles were treated with proteinase K prior to addition of NMIA. SHAPE reactivities for nucleotides (A) 144 to 146, (B) 167 to 170, and (C) 182 to 199, from wt viral RNA are shown. Additional file 4:
**Comparison of SHAPE reactivities for tRNA** ^**Lys3**^ **annealing sequences within Pr + viral RNA from intact (** ***in virio*** **) and proteinase K-treated viral particles (** ***ex virio*** **).** For *in virio* (grey), intact viral particles were treated with NMIA to modify the viral RNA within the viral particle, whereas for *ex virio* (white), the viral particles were treated with proteinase K prior to addition of NMIA. SHAPE reactivities for nucleotides (A) 144 to 146, (B) 167 to 170, and (C) 182 to 199, from wt viral RNA are shown.
