# Supplementary figures and images for: In virio SHAPE analysis of tRNALys3 annealing to HIV-1 genomic RNA in wild type and protease-deficient virus
Source: Retrovirology. 2015 May 16;12:40. doi: 10.1186/s12977-015-0171-7 (PMC4445796; doi:10.1186/s12977-015-0171-7)

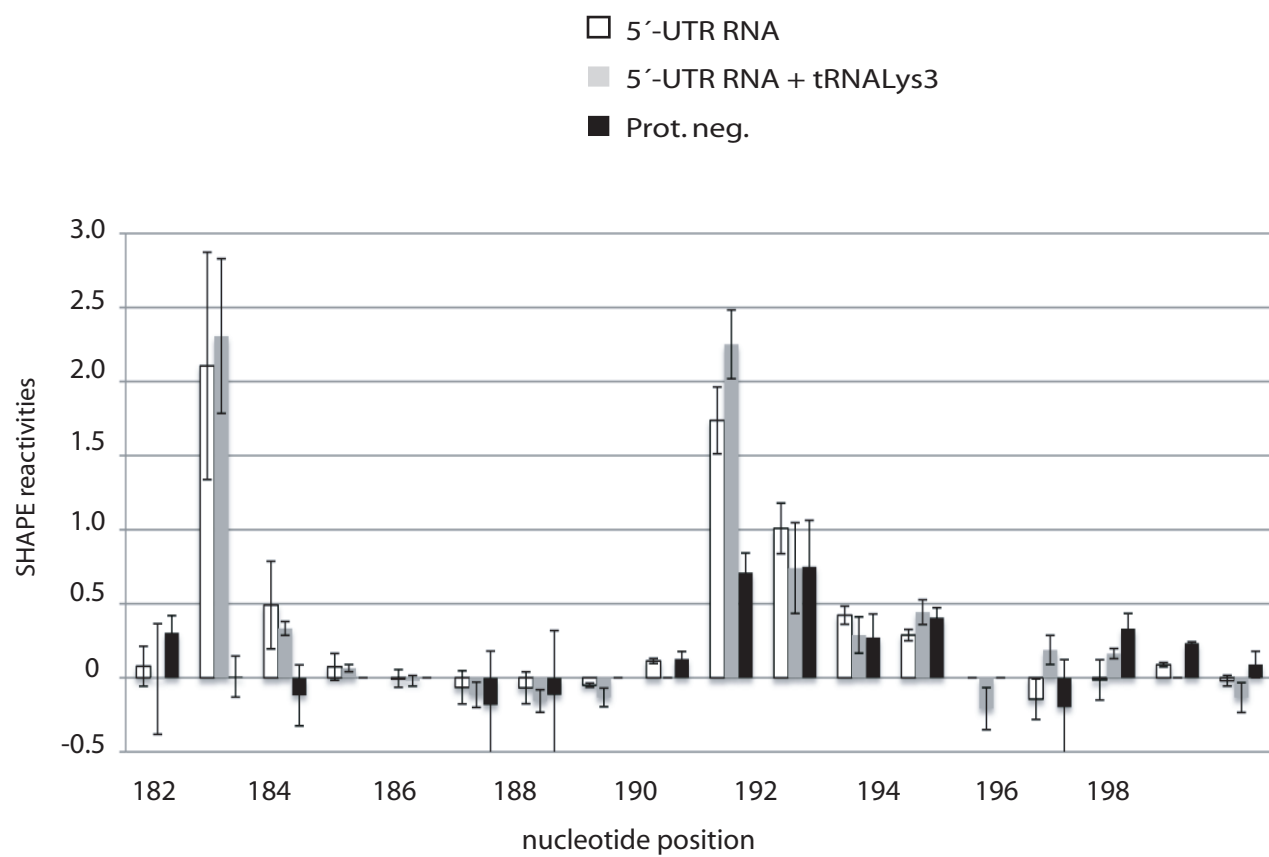

Additional-file-1

Supplement: Additional file 1: — Comparison of SHAPE reactivities for viral RNA nucleotides 182-199 from 5´-UTR RNA, 5´-UTR RNA + tRNA Lys3 , and Pr- virions. SHAPE reactivities for in vitro transcribed unannealed HIV-1 5´-UTR (white), unannealed 5´-UTR + tRNALys3 (grey), and viral RNA found in Pr- (black) viral particles are shown for nucleodtides 182 to 199. The results shown are the averages of three readings at each nucleotide position. The errors shown in the graph correspond to the standard deviations calculated at each nucleotide position. The complete data sets are found in Additional file 2. [file 12977_2015_171_MOESM1_ESM.pdf]

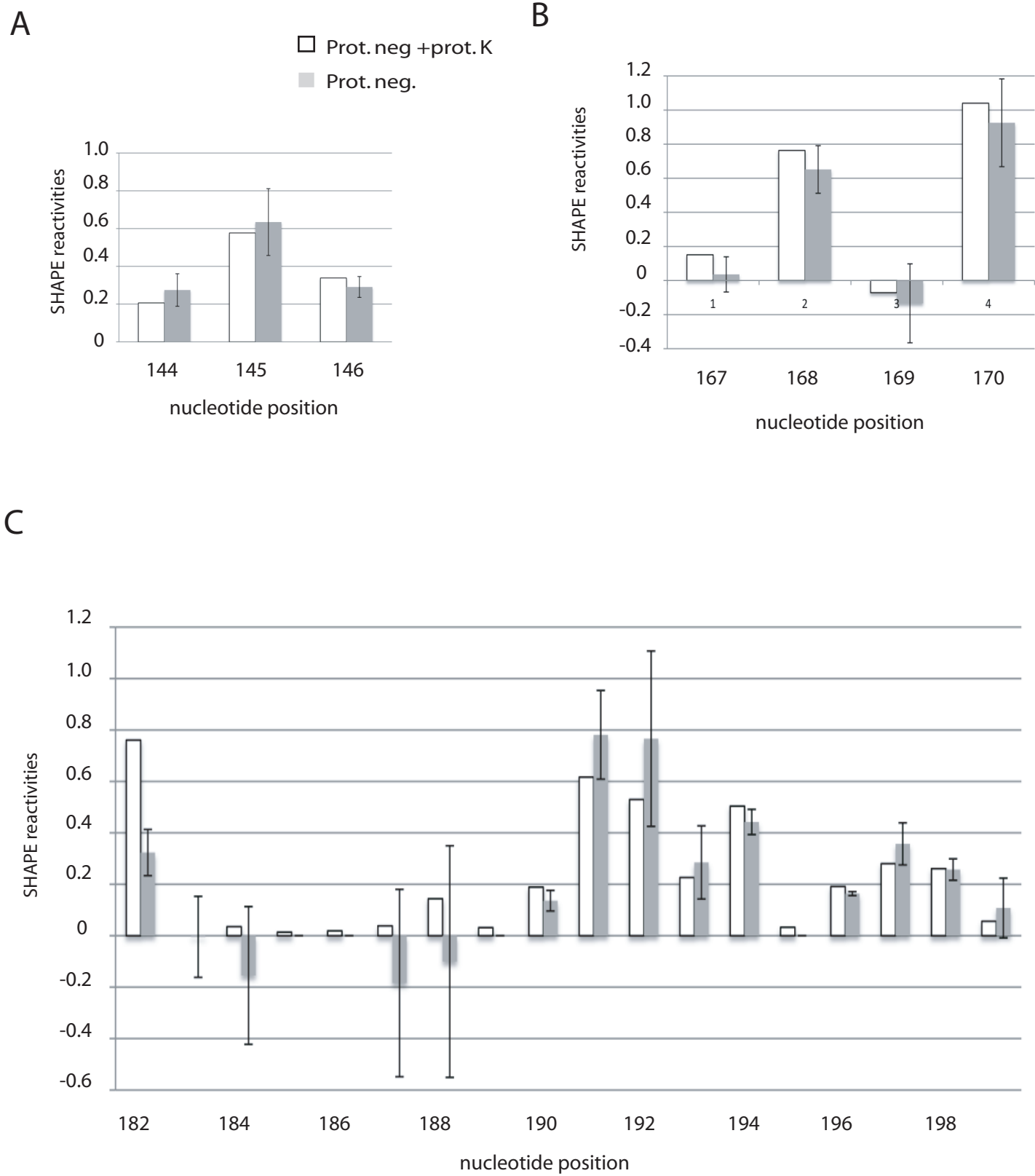

Supplement: Additional file 3: — Comparison of SHAPE reactivities for tRNA Lys3 annealing sequences of Pr- viral RNA from intact ( in virio ) and proteinase K-treated viral particles ( ex virio ). For in virio (grey), intact viral particles were treated with NMIA to modify the viral RNA within the viral particle, whereas for ex virio (white), the viral particles were treated with proteinase K prior to addition of NMIA. SHAPE reactivities for nucleotides (A) 144 to 146, (B) 167 to 170, and (C) 182 to 199, from wt viral RNA are shown. [file 12977_2015_171_MOESM3_ESM.pdf]

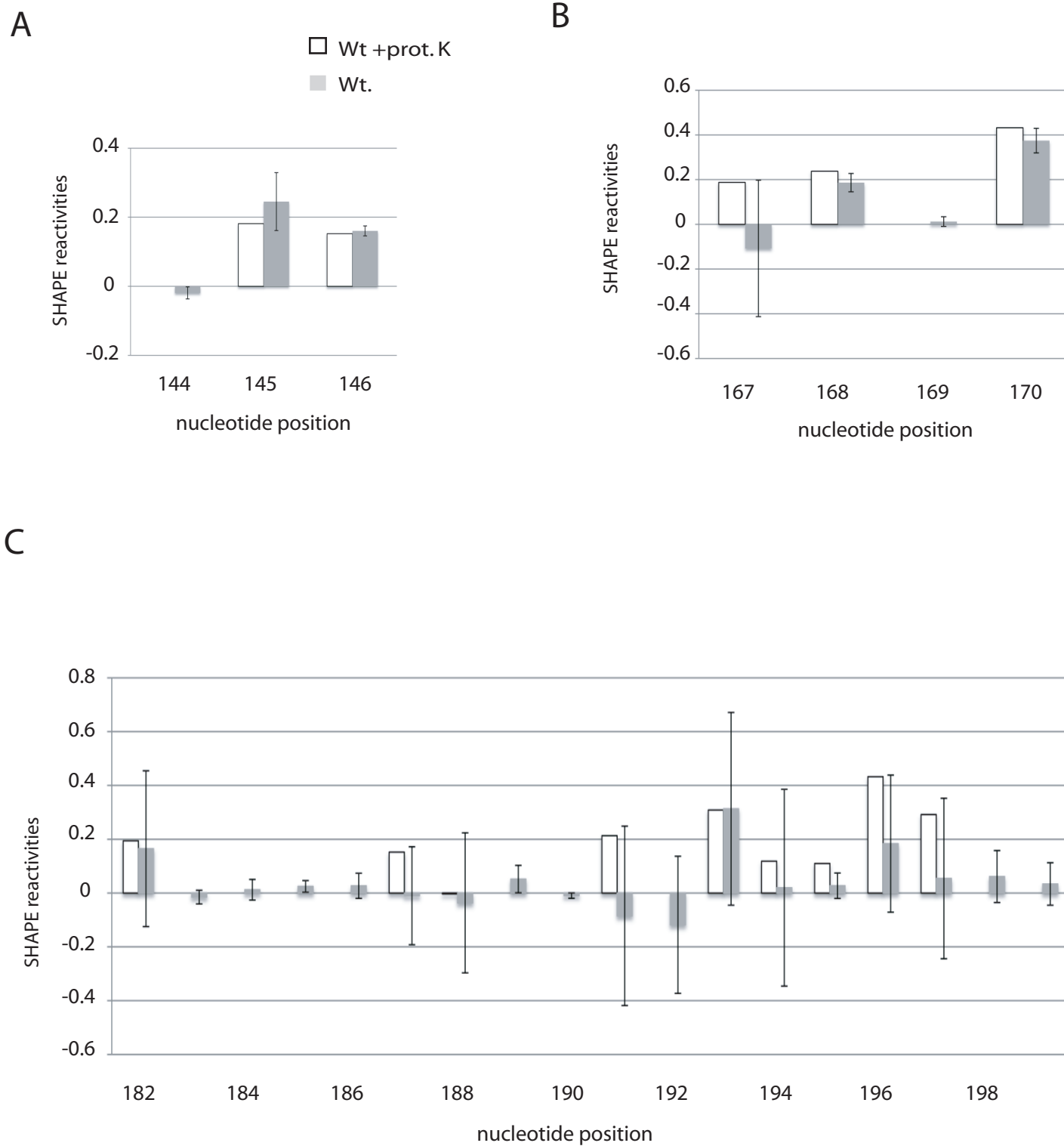

Supplement: Additional file 4: — Comparison of SHAPE reactivities for tRNA Lys3 annealing sequences within Pr + viral RNA from intact ( in virio ) and proteinase K-treated viral particles ( ex virio ). For in virio (grey), intact viral particles were treated with NMIA to modify the viral RNA within the viral particle, whereas for ex virio (white), the viral particles were treated with proteinase K prior to addition of NMIA. SHAPE reactivities for nucleotides (A) 144 to 146, (B) 167 to 170, and (C) 182 to 199, from wt viral RNA are shown. [file 12977_2015_171_MOESM4_ESM.pdf]
